# The theory of mind construct in adulthood: perspective taking in relation to language and executive function

**DOI:** 10.3389/fpsyg.2024.1435685

**Published:** 2024-12-02

**Authors:** Derek E. Montgomery, Virginia Tompkins, Xin Feng

**Affiliations:** ^1^Department of Psychology, Bradley University, Peoria, IL, United States; ^2^Department of Psychology, The Ohio State University at Lima, Lima, OH, United States; ^3^Department of Human Sciences, The Ohio State University, Columbus, OH, United States

**Keywords:** theory of mind, individual differences, perspective taking, early adulthood, theory and measurement

## Abstract

There are conflicting proposals about the underlying structure of the theory of mind (ToM) construct. The lack of clarity impedes attempts to understand relationships between ToM and other cognitive abilities. This study investigated the nature of the ToM construct and its relation to cognitive variables by administering a battery of ToM measurements along with measurements of executive function and general vocabulary to 207 (*M*_age_ = 19.26) adult participants. Associations between ToM tasks were statistically significant after controlling for covariates, but, for the most part, very weak in magnitude. The strongest relationship was between the Strange Stories and Higher-Order False Belief measurements. Previous theoretical analysis proposes those instruments are conceptually linked by a perspective taking requirement that entails representing another’s mental state. Results from a factor analysis suggested an underlying ToM structure—a *protagonist perspective* factor. The Strange Stories, Higher-Order False Belief, and Frith-Happé Animation tasks loaded onto the factor. Its defining feature is the ascription of mental states to predict and explain protagonists’ actions that take place within a narrative structure. It is related more strongly to vocabulary than executive function and it provides grounds for future research on the role of narrative processing in ToM reasoning.

## 1 Introduction

Theory of mind (ToM) is the attribution of cognitive, volitional, and affective states to predict and explain human action. Events that might otherwise appear isolated and incomprehensible are instead integrated within a causal-explanatory network of mental state concepts:

One of the prime functions of everyday belief-desire psychology is to make sense of human actions and minds, to fill in the “becauses” of human life, to answer the everyday “why” questions about what we are doing…. (Wellman, 2014, p. 42).

Research in early childhood focuses on mastery of basic mental state concepts like belief, knowledge, and desire. In story-like scenarios, preschoolers predict and explain what a character will do, or how a character will feel, based upon the character’s mental states (Wellman, 2014). A hallmark of ToM development emerges around age 4 when children master false belief tasks (Wellman et al., 2001). False belief understanding is an index of the capacity to distinguish between an objective state of affairs and a subjective viewpoint about those affairs. Advanced ToM (after age 5) builds upon basic concepts and involves reasoning about misinterpretations and subtle motivations, and making distinctions between what people literally say and what they actually mean (Miller, 2022). Advanced ToM measurements are designed to capture competency in making sense of what someone says or does by integrating context (e.g., situations, personal histories), nonverbal cues, and mental states.

Knowing the adult endpoint of ToM informs research questions for studying its development. For instance, if a mature ToM is fractionated into different subcomponents, then attendant questions ask if the same differentiated structure is present in early ToM and, if so, whether there are different timetables and different underlying cognitive processes for each component. Clarity on the adult endpoint informs hypotheses about cognitive mechanisms underlying its development. There are parallel questions in related areas of developmental psychology. Research on the development of the executive functions (EF) has consistently identified a relatively undifferentiated beginning state in early childhood followed by differentiation into early adulthood (e.g., Karr et al., 2022). There is a rich empirical literature on the adult structure of the EF construct (e.g., Miyake and Friedman, 2012); in contrast, however, there is uncertainty about the structure of ToM in adulthood. We discuss different, conflicting, theories about the structure of ToM in the next section.

### 1.1 The theory of mind construct

There is a growing consensus in the field that the ToM construct, as it is typically defined, is too vague (e.g., Schurz et al., 2021). A three-step process driven by conceptual analysis and research is needed to clarify the construct, its assessment, and, ultimately, the processes that underlie its development (Happé et al., 2017). In the first step, theoretical analysis aims to clarify the construct by “deconstructing” it into criterial subcomponents (e.g., Quesque and Rossetti, 2020; Schaafsma et al., 2015; Schurz et al., 2021). The assumption behind deconstruction is that ToM is not a monolithic construct but, instead, there are varieties of ToM.

Under that assumption, the next steps involve aligning measurements with subcomponents of the construct and, lastly, identifying the neurocognitive processes that underlie task performance.

Taken as a whole, those steps line up the construct, its measurement, and the basic processes driving performance on ToM measurements. In this paper we attempt to advance that research agenda with a focus on the second step. We ask whether widely used ToM instruments cohere in a manner that is consistent with hypothesized subcomponents of the ToM construct. The focus on task coherence naturally leads to considerations about specific cognitive abilities that are common to the tasks that assess a particular subcomponent. We also address, as an attendant consideration in the paper, the relationship between general cognitive abilities and ToM.

The focus of this paper is on the mature form of ToM exhibited by the time individuals reach early adulthood. Although there is considerable research on the coherence of ToM tasks in early to middle childhood (e.g., Osterhaus et al., 2016), comparatively little is known about the coherence of such tasks in adulthood. Extrapolating results from childhood is complicated by the greater variety of task demands for measures of advanced ToM. Early childhood ToM tasks are generally brief and limited to pass-fail response options because of children’s performance limitations (e.g., vocabulary, attention). In contrast, advanced ToM measurements have a much broader range of task demands (e.g., reading vignettes, completing self-report questions, articulating verbal responses to explain characters’ actions, and interpreting ambiguous or subtle perceptual cues). Vast differences in task demands threaten to mask actual coherence among advanced ToM measurements (Devine, 2021). Therefore, advanced ToM tasks represent a stringent test of whether the ToM construct is unitary or coheres into meaningful subcomponents.

The tasks we evaluate are listed in Table 1. We chose them for three reasons. First, they are among the most frequently used instruments in research on advanced ToM (Osterhaus and Bosacki, 2022) with the exception of the newly developed Four-Item Mentalizing Index (FIMI) self-report measurement (Clutterbuck et al., 2021). We added that task to our battery because it targets a competency—cognitive perspective taking—that features prominently in an analysis of the ToM construct discussed below (Quesque and Rossetti, 2020). Second, the tasks in Table 1 (except for the FIMI) have been administered across the lifespan, which means that results from this study are relevant to questions about whether there is developmental continuity in the ToM construct (Apperly et al., 2009). Related, there is variability in performance among adults on the instruments used in this study (e.g., Clutterbuck et al., 2021; Klindt et al., 2017; Launay et al., 2015; Murray et al., 2017; Osterhaus and Bosacki, 2022). Third, the tasks were chosen because they speak to a theoretical point that we elaborate upon in section 1.1.2; namely, that cognitive perspective taking is a defining component of ToM.

**Table 1:** Advanced theory of mind measurements.

*Higher-order false belief* Higher-order tasks assess the understanding that characters in scenarios can have false beliefs about beliefs (e.g., Perner and Wimmer, 1985). Higher-order tasks recursively embed desires and beliefs such as A wanted B to know that A believes… Questions range in complexity, with up to seven levels of embeddedness. The participant’s perspective counts as the first level; thus, level-two embeddedness is about one mental state (e.g., “A wanted X”), level-three would be, “A thought that B wanted X,” and so forth (Launay et al., 2015).
*Reading-the-mind-in-the-eyes test (RMET)* Photographs of the eye region depict expressions of emotion and thought. Participants choose a mental state descriptor (e.g., *suspicious*) from among four options that best describes what the person is thinking or feeling (Baron-Cohen et al., 2001; Olderbak et al., 2015).
*Frith-Happé animation* A brief video displays geometric shapes moving in a manner that suggests both contingency and narrative elements such as goals and emotional reactions. Participants’ descriptions of what happened in the scenes are coded for mention of thoughts, desires, emotions, and other ToM-related constructs (Abell et al., 2000).
*Strange stories* A series of brief scenarios portray social interactions that feature a non-literal verbal utterance (e.g., a deceptive or ironic statement). Participants are asked to interpret the utterance and they are scored on whether they infer the speaker’s intended meaning underlying the literal content of the utterance (White et al., 2009).
*Four-item mentalizing index (FIMI)* A self-report measurement of the ability to infer the cognitive perspective of others (Clutterbuck et al., 2021).

In order to gain clarity on the structure of the construct and the processes that support it, we address three questions in this paper:

- Are there interrelations among some (or all) of the ToM measurements in Table 1?
- If there are interrelations, do they cohere into theoretically meaningful clusters (subcomponents) or are the relations sufficiently widespread to suggest ToM is a unitary construct?
- If there are interrelations, are they related to general cognitive (including language) skills and/or socioeconomic status?

Below, we expand upon questions a, b and c.

#### 1.1.1 Are there interrelations among ToM measurements?

In principle, it is reasonable to expect that, all else equal, measurements of ToM might show at least some degree of interrelatedness despite differences in the tasks’ demands, formats, and modalities. This is because mental state concepts of belief, desire, intention, perception, and emotion cohere to form a causal-explanatory network within the ToM construct (Wellman, 2014).

To illustrate, in false belief tasks an agent sees the location of an object but does not witness its transfer to another location (e.g., Wimmer and Perner, 1983). Participants infer where the agent will search for the object upon returning to look for it. Within that brief scenario, there is a complex interplay among the concepts of perception, belief, desire/volition, and emotion. The agent’s desire to find the object motivates the search for it; however, the nature of the search (where the agent looks) is mediated by the belief about its location. The belief about its location is, in turn, tied to the agent’s earlier perceptual experience. The resultant surprise and disappointment upon looking in the wrong location are tied to the agent’s desire and false belief. Thus, while task content and demands will vary, “solutions” to the tasks will draw from the same causal-explanatory framework to make sense of the agent’s actions by ascribing interrelated mental state concepts. In that sense, ToM is a unitary construct, leading one to expect a widespread degree of relatedness between diverse measures of ToM (see Warnell and Redcay, 2019 for a discussion).

At the other end of the continuum, Warnell and Redcay (2019) found that ToM tasks were unrelated to one another in adulthood (effect sizes ranged from *r* = −0.115 to *r* = 0.125). Their results suggest that ToM has a diverse, and perhaps even fragmented, structure. While instructive, there are limitations to the Warnell and Redcay study. First, their task choice prioritized identifying a diverse array of measurements, but it was not theoretically driven. They acknowledged in the discussion of their findings that, “future studies should employ a targeted set of tasks in order to test specific underlying structures of social cognition” (p. 8). Second, they did not include measurements of EF, general language skills, or SES as covariates in their adult sample. The inclusion of those constructs is important for both theoretical and methodological reasons, as discussed in section 1.1.3.

#### 1.1.2 Perspective taking and theory of mind

The tasks in our study are different versions or differ entirely from those used in Warnell and Redcay (2019). With respect to task variants, both studies used RMET and the Higher-Order False Belief tasks, but the studies used different versions of the tasks (see section 2). The task choice in the present study is theoretically motivated, with a focus on a recent analysis of the ToM construct by Quesque and Rossetti (2020), which we discuss in this section. In particular, we ask if ToM measures cohere into a meaningful *perspective taking* component.

Meta-representation, the “conceptual heart” of ToM, involves (a) representing the content of others’ mental states (i.e., their perspectives) and (b) doing so even when perspectives are in conflict between individuals and/or with reality (Rakoczy, 2022, p. 1). Quesque and Rossetti (2020) argued that those criteria are inconsistently assessed in tasks that purportedly measure ToM. In their analysis, some tasks, such as the Strange Stories and False Belief tasks, meet both criteria (Quesque and Rossetti, 2020). The FIMI, as a measure of individual differences in cognitive perspective taking, would presumably relate to both measurements. Other tasks, such as those that involve the recognition of mental states from perceptual stimuli, do not meet the criteria. The RMET and Animation tasks both fall into the latter category. Performance on those measurements, they argue, involves low level perceptual processes of recognition and categorization rather than actually representing the conflicting perspectives of different agents.

Meinhardt-Injac et al. (2020) found evidence for a two-component model of ToM in a study of participants between 11 and 25-years-old. The social-perceptual component entails inferring mental states from nonverbal cues (such as the eyes). Mental state judgments are immediate and directly elicited from perceptual stimuli. The social-cognitive component involves verbal reasoning about mental states. In distinguishing between social-perceptual and social-cognitive components, the two-factor model is consistent with Quesque and Rossetti (2020).

The Quesque and Rossetti (2020) analysis suggests that the Strange Stories and False Belief tasks (and the FIMI) should be related to one another, but not related to the Animation and RMET tasks. However, there is inconsistent support for that prediction within the childhood literature. On the one hand, measurements that should be related are, in fact, not always related. For instance, Hayward and Homer (2017) found that Second-Order False Belief and Strange Stories tasks were unrelated among 7–13-year-olds (*n* = 107; *r* = 0.06). Also, measurements that should be unrelated in the Quesque and Rossetti analysis sometimes actually are related. Devine (2021) suggests that the Animation and Strange Stories tasks share the same conceptual core. In both instances, participants explain social behavior by inferring the mental states of agents. In support of that characterization, the tasks were significantly correlated in 10-year-old children (*n* = 137; *r* = 0.34) and loaded onto a single latent factor (Devine et al., 2016; see also, Lecce et al., 2021).

Partial support comes from factor analytic work by Osterhaus et al. (2016). They found that three measurements—Higher-Order False Belief, Strange Stories, and the RMET—cohered into a social reasoning factor for children in grades 2–4 (*n* = 466). Two other factors were reasoning about ambiguity and recognition of social norm transgressions. The relationship between Higher-Order False Belief and Strange Stories is at odds with Hayward and Homer (2017). One explanation is task variation between studies. For instance, Hayward and Homer reported comprehension problems for the Strange Stories task in their sample. Consequently, they limited their analyses to just a subset of the items for which children passed comprehension questions. More generally, in the ToM literature there are multiple task variants, which complicates between-study comparisons and likely contributes to inconsistent findings.

Inconsistencies in the literature may also stem from different age ranges found between samples. There was a narrower age range in Osterhaus et al. (2016) than in Hayward and Homer (2017). In general, the narrower the age range, the less likelihood there is of conflating developmental and individual differences (see Devine, 2021). The problem with a large range is that variability in performance on a particular task might be minimal for children at the low or high age range of a sample. For instance, children at the low range of the sample may have a conceptual deficit that severely restricts performance relative to children at the upper age range of the sample. Alternatively, children in the upper age range may have reached ceiling on a task.

Although comparisons between studies are difficult for the reasons given above, the point that is most germane to the present study is simply that there is a need to empirically test the Quesque and Rossetti (2020) analysis because support is not unequivocal in the literature. The patchwork of findings highlight the need for research that will inform whether the Quesque and Rossetti analysis actually captures the mature form of the ToM construct. In particular, there is a need for research that tests ToM performance on a battery of widely used measures administered to a large adult sample that has a restricted age range. A narrow age range, for the reasons noted above, “is essential for studying individual differences in ToM and for examining the relations between tasks*”* (Devine, 2021, p. 61).

#### 1.1.3 Cognitive influences

Studies of adult ToM that are focused on relationships with language and EF are uncommon in the literature (Miller, 2022). Even when studies of advanced ToM do include measures of EF and language, Miller points out that frequently the goal is to confirm equivalency between groups rather than investigate their influence on ToM. A further complication for understanding how ToM is related to language and EF in adulthood stems from the tendency to analyze ToM with a composite score. Doing so limits our understanding of how general cognitive influences might vary across different measures. Put another way, there is an incomplete picture of how language and EF relate to performance on a range of different measures of advanced ToM in adulthood.

Osterhaus and Bosacki (2022) found that general language skills (mostly vocabulary) and EF (inhibition) were related to individual differences in advanced ToM in their analysis of studies across the lifespan. They calculated the average correlation by collapsing across tasks and age groups. Both correlations were statistically significant, but the magnitude of the relation was greater between language and advanced ToM (*r* = 0.376) than for inhibition (*r* = 0.152). Because Osterhaus and Bosacki (2022) included a wide age range in their analyses, their findings do not speak directly to the potential influence of cognitive variables on advanced ToM in adulthood. Nevertheless, the results indicate a potentially consequential role for both variables and warrant inclusion of both language and EF as covariates in the present study.

Accounting for language and EF in the present study allows us to address a theoretical question surrounding their respective roles in adults’ ToM. Language and EF are associated with ToM development in early childhood (e.g., Tompkins et al., 2019; Devine and Hughes, 2014). The variables are posited to promote both the emergence and expression of ToM in childhood (Carlson et al., 2013). *Expression* refers to task performance; for instance verbal tasks require general language skills for comprehending scenarios and test questions. *Emergence* refers to a role for language and EF in producing variation in the timing of conceptual development. For instance, preschoolers’ understanding of complement syntax supports the development of false belief understanding (de Villiers, 2021). Language in that case supports the development of a core ToM concept.

Core concepts of belief and desire are in place prior to advanced ToM. Therefore, it is unlikely that variability in task performance on advanced ToM tasks would be due to conceptual deficiencies *per se*:

“Thinking in terms of mindreading concepts does not help us understand variability of mindreading in typical adults, because there is no variance in the possession of such concepts beyond late childhood” with Apperly and Wang (2021, pp. 100–101).

Variability in advanced ToM reflects individual differences in the fluency of social reasoning and in sensitivity to subtle social cues rather than mastery of basic concepts. Advanced ToM tasks are not designed to capture conceptual deficits in an all-or nothing (pass-fail) manner; instead, the tasks generally measure the consistency with which conceptual knowledge is applied to problems in social interactions. From that perspective, it seems more likely that language and EF, if they are responsible for individual differences in advanced ToM, would have an impact on the expression of ToM reasoning instead of the development of new conceptual knowledge (Apperly, 2021).

The question, then, is whether the influence of language and EF in the development of conceptual knowledge in childhood endures in the expression of that knowledge in adulthood, or whether linguistic and EF skills have reached a level of proficiency by early adulthood that is sufficient for mastering advanced ToM tasks (Apperly, 2021). In the latter case, language and EF would no longer be limiting factors in task performance and, therefore, would be unrelated to individual differences in ToM. Evidence consistent with the latter possibility comes from a recent meta-analysis of individual differences in EF and second-order false belief understanding (Peloquin et al., 2023). The magnitude of the relationship between EF and second-order false belief decreased with age, and by adulthood it was non-significant. However, with one exception, the studies only employed a second-order false belief task, and that task is subject to ceiling effects in adulthood (Osterhaus and Bosacki, 2022). The false belief measurement in our study (Launay et al., 2015) includes higher-order questions that recursively embed mental states beyond the second order (see Table 1). As noted earlier, we deliberately chose that task because it produces variability in adult samples (Launay et al., 2015).

A third variable of interest is the socioeconomic status (SES) of the participants. SES is a common covariate in ToM research in childhood, and there is evidence that it is related to ToM development (e.g., Tompkins et al., 2017; see Devine and Hughes, 2018 for a meta-analysis). To be clear, SES itself is not a cognitive variable, but it is a proxy for a wide range of variables that include education and parental behaviors associated with children’s language and cognitive development. Longitudinal evidence indicates that SES differences in early childhood are associated with educational status and general cognitive skills in young adulthood (Osler et al., 2013). Consequently, it is possible the SES-ToM relationship observed in childhood may extend into early adulthood. However, there is relatively little research examining that possibility.

In sum, the first research question for this study asks if different measurements within a ToM battery are interrelated even when accounting for covariates. If the ToM construct is unified, then commonly used measurements of the construct should be interrelated. If the ToM construct has an unstable structure, then tasks should generally be unrelated because there is not a single, foundational core component common to all (e.g., Warnell and Redcay, 2019). The second question asks if factor analysis reveals evidence of coherence among the tasks into a theoretically meaningful factor or factors. The factor would represent a subcomponent of ToM. One candidate subcomponent is a “perspective taking” factor that would include Higher-order False Belief, Strange Stories, and the FIMI tasks (Quesque and Rossetti, 2020). Finally, if evidence indicates the presence of a factor, a third question asks how the factor is related to other cognitive variables. Individual differences in ToM in childhood are associated with vocabulary and EF. If the influence of those variables is continuous, then we would expect them to also bear a relation to individual differences in adulthood (see Apperly, 2021).

## 2 Methods

### 2.1 Participants

Undergraduate students were recruited from Introductory Psychology courses on two college campuses in the Midwest United States. The study received Institutional Review Board approval from The Ohio State University; written consent was obtained for all participants; and participants received course credit for their participation. Participants (76% female) included 207 adults (*M*_age_ = 19.26, *SD* = 1.13, range = 18.02–26.69); 49 identified as male, 158 as female, and no participants identified as non-binary or chose to self-describe. The sample size is sufficient for uncovering lowest practically meaningful correlation coefficients (*r* = 0.20). The race of participants was 74% White, 15% Black, 7% Asian, 3% Other, and < 1% American Indian or Alaska Native; assessed separately, 12% also identified as Hispanic or Latino. Race and ethnicity closely mirror proportions in the U.S. population (U.S. Census Bureau, 2022).

### 2.2 Measures

#### 2.2.1 Theory of mind

*Reading the mind in the eyes.* This task presents four sets of eyes and participants choose the mental state that best matches the expression in the eyes. We used the shortened version of 10 sets of eyes (Olderbak et al., 2015) because it has better internal reliability than the original 36 sets of eyes (Baron-Cohen et al., 2001) used in the Warnell and Redcay (2019) study. Pages (10 plus one practice item) with eyes and four answer choices were presented in a binder and participants recorded their responses on an answer sheet. For example, for the target mental state “pensive,” the other three answer choices were *irritated*, *excited*, and *hostile*. Definitions of all answer choices were provided in the binder. We calculated Kuder–Richardson 20 (KR20) for internal reliability for the eyes task given its binary scoring (0 or 1), which was 0.31.

*Frith-Happé animation.* The Animation task (Abell et al., 2000; White et al., 2011) presents participants with a big red triangle and a small blue triangle moving about a white background. Animations varied in length between 34 and 45 s and were presented on an iPad. We administered one practice animation and the four ToM animations (coaxing, mocking, seducing, surprising). To illustrate, in one of the animations scenarios a big triangle and a small triangle interact in a manner that suggests one is coaxing the other to come out of a box-shaped room. At the end of each animation, participants were asked “What was happening in this animation?.” Responses were recorded and transcribed verbatim. Each response was coded for the level of intentionality according to Lecce et al. (2021). Scores ranged from 0 to 5 on each animation for a total of 20 possible points; 20% of responses were coded for interrater reliability, which was 87.5%. We report McDonald’s omega as it tends to be more robust than Chronbach’s alpha when scales might have responses that cluster around one end. Internal reliability was *ω* = 0.64 in our sample.

*Strange stories.* The Strange Stories task presents brief vignettes that describe a social or physical event that participants are asked to explain (White et al., 2009). We focused on the eight social stories because they are designed to test the ToM construct. These stories describe interactions that feature a non-literal verbal utterance (e.g., a joke or an ironic statement). Participants read the scenarios in a binder followed by the test question by the experimenter, often asking why a character would say something (e.g., white lie). Responses were recorded and transcribed verbatim. Scoring followed White et al. (2009) and ranged from 0 to 2 for each story for a total of 16 possible points; 20% of responses were coded for interrater reliability, which was 92%. Internal reliability was *ω* = 0.48 in our sample.

*Higher-order false belief:* In *An Imposing Memory Task* (Launay et al., 2015) participants read three short stories and then answered a series of true/false questions containing both factual recall and mentalizing questions, which vary from two to seven levels of embeddedness (the reader is considered level one). The task avoided ceiling effects because it included recursive reasoning beyond the second-order. For instance, one of the stories described a situation that required third-order reasoning. After reading the story, participants were asked whether it was true or false that one character thought that another character (Sam) was wrong in thinking a third character wanted to trick Sam. We were interested only in the mentalizing questions (10 per story for a total of 40 questions). Participants read and answered the questions in a Qualtrics survey. The Warnell and Redcay (2019) measurement, in comparison, contained 45 second and third-order ToM questions (adapted from Kinderman et al., 1998). We calculated Kuder–Richardson 20 (KR20) for internal reliability for the false belief task given its binary scoring (0 or 1), which was 0.69.

*Four-item mentalizing index:* This index was developed to assess self-reported mentalizing (i.e., non-emotional mental states; Clutterbuck et al., 2021). It included four statements, for example, *I find it easy to put myself in someone else’s shoes*. Participants responded on a 4-point scale ranging from strongly disagree (1) to strongly agree (4) for a total of 16 possible points. Internal reliability for the four item mentalizing index is good; Clutterbuck et al. (2021) reported *ω* = 0.75 and *ω* = 0.70 in our sample. Given its questionnaire format, this task was also presented in the Qualtrics survey.

#### 2.2.2 Covariates

*Picture vocabulary test:* Participants completed the Picture Vocabulary Test (PVT) using the NIH Toolbox application on an iPad. This task requires participants to select the picture (out of four) that best matches the word spoken. The PVT is an adaptive test; items differ across participants depending on their performance. Participants see a maximum of 25 items and administration stops when participants reach a standard error of less than 0.03. This measure has good test-rest reliability (*ICC* = 0.80) and converges with another measure of receptive vocabulary (*r* = 0.80) in a sample of adults up to the age of 85 (Gershon et al., 2014). Given the restricted age range of students in our study, age was unrelated to the PVT and uncorrected standard scores were used in analyses.

*Executive function:* Participants completed three executive function tasks from the NIH Toolbox application. In the Flanker Test, a measure of inhibitory control and attention, participants must focus on a directional stimulus in the center of the screen and respond with the correct direction (left/right) while inhibiting the stimuli flanking it. In the Dimensional Change Card Sort Test, a measure of cognitive flexibility and attention, participants must sort by both color and shape and correctly shift their attention to the current rule (color/shape). Finally, in the working memory task, participants must remember and sequence objects (animals or food) presented visually and via audio. In the 1-list version, participants see and hear a random sequence of either animals or food and must repeat them back in the order of their size. In the 2-list version, participants must repeat back both animals and food, sorting first by category then by size. Difficulty increases by 1 item (with two trials for each number of items) until participants incorrectly sequence both trials.

Scores in the Flanker and Dimensional Change Card Sort Test reflect both accuracy and reaction time given that adults tend to be highly accurate but slow down their reaction time to improve their accuracy. A log base 10 transformation is used to normalize the distribution of scores given that they tend to be positively skewed. This two-vector scoring allows interpretation of adult scores on these tasks in relation to performance of younger children (see Zelazo et al., 2014, for details). Scores in the working memory task reflect the number of correct items, transformed to a standardized score (Tulsky et al., 2014). The NIH Toolbox Flanker Test and Dimensional Change Card Sort Test (Zelazo et al., 2014) and working memory test (Tulsky et al., 2014) have been validated in adults (aged 20–85 years); they demonstrate high test–retest reliability and concurrent validity with other measures of executive function. Performance on these tasks tends to peak in early adulthood and decline in the later decades of life (Tulsky et al., 2014; Zelazo et al., 2014). Thus, these tasks are appropriate in adult samples. Given the restricted age range in the current sample, participant age was unrelated to any of the executive function measures and uncorrected standard scores were used in analyses.

*Socioeconomic status:* Because of the homogeneity of college students’ education level, participants reported their parents’ highest level of education on an 8-point scale ranging from less than high school degree (1) to professional degree (8); education level was averaged for students raised by two parents. Participants also reported their subjective social standing using the MacArthur Scale of Subjective Social Status which presents a ladder with 10 rungs in which 10 represents those with the most money, most education, and best jobs (Adler et al., 1994). This item was also presented in the Qualtrics survey. Z-scores of parents’ average education level and subjective social status were summed to create an SES variable.

### 2.3 Procedure

Participants completed all assessments during two sessions in a university laboratory space one-on-one with a trained researcher. Informed consent was obtained during the first session followed by the Qualtrics survey (demographics, SES, the Higher-Order False Belief and FIMI tasks). The covariates were then administered on an iPad. The remaining ToM tasks (RMET, Strange Stories, and Animation) were completed at the second session.

### 2.4 Missing data

Eleven participants (5%) did not return for the second day of testing and so are missing day 2 measures (RMET, Strange Stories, and Animation). A few other variables are missing one to two participants due to experimenter error or noncompletion by the participant; thus, sample sizes range from 195 to 207 depending on the variable. All analyses were repeated with imputed data sets to utilize the full sample; all results were the same. Thus, results are presented with collected data only, not imputed data.

## 3 Results

Table 2 provides the descriptive statistics for all covariates and ToM measures. There was a wide distribution of parents’ education; 20% of parents had a high school diploma or less; 35% had some college or an associate’s degree; 28% had a bachelor’s degree; and 17% had a graduate degree. The median (4.00) corresponds to an associate’s degree. The mean for subjective social status was 6.13 (out of 10) and responses ranged from 1 to 9. There were no significant gender differences on any ToM measure (*p*s > 0.33) and so gender was not controlled in subsequent analyses. Despite the adult age group, there was wide variability on all ToM measures, demonstrating that the ToM skills tested were not at ceiling.

**Table 2:** Descriptive statistics for study variables.

Variable	*N*	Mean	SD	Range
Covariates				
Parent education	206	3.93	1.46	1–8
Subjective social status	207	6.13	1.58	1–9
Picture vocabulary test	205	99.16	6.96	76–118
Flanker	205	103.83	9.38	81–198
Dimensional change card sort	205	111.12	6.72	86–120
Working memory	205	106.34	9.85	78–136
Theory of mind				
Reading the mind in the eyes	196	7.32	1.59	3–10
Animation	196	12.88	3.73	2–20
Strange stories	195	12.12	2.27	5–16
Higher-order false belief	206	26.07	3.07	15–30
Four-item mentalizing index	207	12.83	2.09	6–16

Next, we examined the bivariate correlations among all covariates and ToM measures (Table 3). Correlation coefficients with the covariates partialled out are in parentheses. Age and SES (i.e., the composite of parents’ education and subjective social status) were unrelated to participants’ ToM abilities. However, receptive vocabulary (PVT) was significantly and positively related to RMET, Strange Stories, and Higher-Order False Belief Understanding. Both of the ToM perceptual measurements (RMET and Animation) were related to a single EF component; otherwise, there were no relations between EF and ToM.

**Table 3:** Bivariate and partial correlations among covariates and ToM.

	Age	SES	PVT	Flanker	DCCS	WM	RMET	AN	SS	FB	FIMI
Age	–	−0.06	0.13	−0.02	0.09	−0.05	0.00	0.02	−0.04	0.06	−0.08
SES		–	0.09	0.15*	0.09	0.08	−0.04	0.01	0.11	−0.10	0.08
PVT			–	0.08	0.18*	0.29***	0.25**	0.10	0.22**	0.25***	−0.12
Flanker				–	0.49***	0.07	0.15*	0.11	0.00	0.08	0.00
DCCS					–	0.13	0.04	0.17*	0.08	0.13	0.02
WM						–	0.07	0.12	0.09	0.08	−0.22**
RMET							–	0.07(0.06)	0.08(0.06)	0.11 (0.05)	−0.03(0.01)
AN								–	0.18*(0.15*)	0.19**(0.17*)	0.16*(0.19*)
SS									–	0.27***(0.26***)	0.10(0.12)
FB										–	0.10(0.14*)
FIMI											–

***p < 0.001, **p < 0.01, *p < 0.05. SES, socioeconomic status; PVT, Picture Vocabulary Test; DCCS, Dimensional change card sort; WM, Working memory; RMET, Reading the mind in the eyes test; AN, Animation; SS, Strange stories; FB, False belief; FIMI, Four-item mentalizing index. Correlations in parentheses control for age, SES, PVT, Flanker, DCCS, and WM.

We next examined the factor structure of the five ToM tasks. We conducted an exploratory factor analysis, suppressing coefficients <0.40. Bartlett’s test of sphericity was significant (35.39, *df* 10, *p* < 0.001). The Kaiser-Meyer-Olkin measure of sampling accuracy was 0.62. Two factors were identified based on eigenvalues (>1), total variance explained (52%), and examination of the scree plot. The eigenvalues for the first two factors were 1.54 and 1.03. The component matrix is presented in Table 4, showing that Animation, Strange Stories, and Higher-Order False Belief formed one factor, whereas Reading the Mind in the Eyes and the Four-Item Mentalizing Index formed a second factor but one in which the FIMI was negative.

**Table 4:** Factor loadings of ToM measures.

ToM measure	Factor 1	Factor 2
Reading the mind in the eyes		0.73
Animation	0.62	
Strange stories	0.67	
Higher-order false belief	0.69	
Four-item mentalizing index		−0.67

Finally, we examined Factor 1 in relation to the covariates. A composite of the z-scores for Animation, Strange Stories, and Higher-Order False Belief was significantly related to receptive vocabulary (*r* = 0.28, *p* < 0.001), cognitive flexibility (*r* = 0.19, *p* < 0.01), and working memory (*r* = 0.14, *p* < 0.05), and unrelated to age, SES, or inhibitory control (all *p*s > 0.23).

## 4 Discussion

There is a need in the field for (a) mapping out the subcomponents that comprise the ToM construct and (b) determining if broadly used ToM tasks are measuring particular subcomponents of the ToM construct. Conceptual analysis is one approach toward addressing that need. Quesque and Rossetti (2020) identified a core component of the ToM construct—cognitive perspective taking—and then conceptually distinguished between widely used measurements that do or do not assess that core dimension. A second, complementary, approach in mapping out subcomponents is empirical in nature, and that is the one taken in this study.

This study investigated whether there is task coherence in early adulthood that maps onto the core cognitive perspective taking dimension posited by Quesque and Rossetti (2020). Couched within that question, we investigated the influence of individual differences in vocabulary, EF skills, and SES (which is a proxy for general cognitive abilities) on advanced ToM measurements. The results of the study carry four implications for our understanding of the ToM construct.

First, for the most part the correlations between different ToM measurements were very weak. That finding indicates ToM is not a unitary construct; in other words, there is not a single dimension of ToM that is captured across a spectrum of ToM instruments. Given the breadth of the construct, we suggest researchers avoid relying on a single measurement for advanced ToM and instead use a battery of instruments. Related, we also suggest that investigators consider examining different advanced ToM measurements separately in their analyses rather than forming a single composite that would obscure meaningful differences in what the instruments are measuring.

There is a caveat when interpreting the correlations. The internal consistency of the ToM measures was not strong, overall, in this study. That result is consistent with findings in samples of children and adolescents (e.g., Hayward and Homer, 2017; Osterhaus et al., 2016). The weak correlations could reflect, to some extent at least, the psychometric limitations of the instruments themselves. All else equal, low reliability reduces the likelihood of finding relations between instruments.

Second, with respect to cognitive influences, our results complement those found in the childhood research literature in that we found a stronger relationship between language and ToM than for EF and ToM (see Osterhaus and Bosacki, 2022). For the most part, measurements of three EF components were unrelated to ToM (see Table 3). It could be the case that EF skills have reached a level of maturity by early adulthood sufficient for mastering ToM task demands. Alternatively, it could be that ToM tasks with higher processing demands, such as tasks that use response time as a dependent variable, are more sensitive to EF differences than the untimed tasks administered in this study (see Apperly, 2021 for a discussion). A third possibility is that a different approach to measuring EF could result in a different finding. For instance, we assessed three different components of EF, but it could be that a battery of tasks focused on a single component (e.g., inhibition) yields a more robust link between ToM and adult EF than the one reported in this study. In sum, we have contributed evidence that speaks to the relation between EF and ToM in adulthood, but more research is needed to understand the magnitude and qualities of that relationship.

In contrast to EF, there is a clearer case for the relevance of language for ToM. As with most studies on advanced ToM, we used a measurement of receptive vocabulary to assess language skills (Osterhaus and Bosacki, 2022). We found weak, but non-negligible, relations between vocabulary skills and performance on tasks that place demands on verbal comprehension skills (see Table 3). We briefly elaborate on those relationships.

The Strange Stories and Higher-Order False Belief tasks each draw upon text comprehension skills for processing narratives in the tasks. Text comprehension is related to vocabulary knowledge (e.g., Ouellette, 2006). The association between the RMET and language (0.19 in magnitude) is worth noting because the RMET is often classified as a perceptual task. However, task performance also requires vocabulary knowledge of the various emotion and cognitive terms in the task (e.g., *pensive*). The inclusion of a glossary of definitions for the response options in the task reflects its relatively challenging vocabulary demands (see Miller, 2022). Considered as a whole, the results suggest that the relation between language and ToM observed in early childhood (see Milligan et al., 2007 for a meta-analysis) may be present in early adulthood, perhaps in a weakened form, on tasks that place a demand on verbal comprehension (vocabulary and reading). Returning to a question raised in the Introduction, the results suggest that even after basic language skills have reached maturity in early adulthood, individual differences may still relate to performance. As discussed earlier, the *expression* of advanced ToM knowledge on verbal tasks may vary in relation to vocabulary skills.

Third, if ToM is not a unified construct that demonstrates coherence among diverse measurements, is there evidence for a dimension, or subcomponent, of the construct? We found evidence that suggests different measurements overlap in assessing a dimension of the construct. The relation between Strange Stories and Higher-Order False Belief exceeded the threshold for a practically meaningful correlation in this study. The association was significant even after accounting for SES, language, and EF. The tasks are the two most widely used measurements of advanced ToM (Osterhaus and Bosacki, 2022), and their association is telling because they are highly representative of how the advanced ToM construct is operationalized in research. Recent effect size guidelines, including an analysis of over 700 meta-analytically derived correlations, characterize correlations between 0.2 and 0.3 as “typical” and “medium” effect sizes (Funder and Ozer, 2019; Gignac and Szodorai, 2016). We do not want to overstate the relation between Strange Stories and Higher-Order False Belief, but we propose that the relationship between them is theoretically meaningful.

In particular, the connection between Strange Stories and Higher-Order False Belief is consistent with Quesque and Rossetti (2020, p. 386) who argued that both instruments capture a core dimension of ToM: The “ability to corepresent—or to switch between—different perspectives.” In essence, we found that (a) two instruments highly representative of the advanced ToM construct and theorized to tap into the perspective taking dimension of ToM are, in fact, related and (b) the relation is not due to shared covariance with other cognitive influences.

Fourth, unlike factor analytic results from Warnell and Redcay (2019), our results indicated an underlying ToM structure for advanced ToM. Two factors emerged in this study (see Table 4), but the second factor (RMET and FIMI) is uninterpretable. The RMET and FIMI were unrelated (see Table 3) and do not bear a clear conceptual connection because the FIMI was developed to measure the “understanding of non-emotional mental states” (Clutterbuck et al., 2021, p. 2). Although the FIMI and RMET were related in the Clutterbuck et al. study, Murphy et al. (2022) point out that if the RMET is primarily measuring empathy and emotion perception, then it should actually be unrelated to the FIMI. The findings in the present study are consistent with that interpretation. The age range (18–78-years) in Clutterbuck et al. (2021) was substantially different from the more restricted range in this study. ToM proficiency changes throughout the lifespan (e.g., Miller, 2022), and it could be that there is a developmental explanation for the different results.

Factor 1, however, is interpretable based upon previous evidence and theory. The Strange Stories, Higher-Order False Belief, and Animation tasks formed that factor. The inclusion of the Strange Stories and False Belief measures within a single factor supports claims that both instruments measure perspective taking about cognitive states (Quesque and Rossetti, 2020). The inclusion of the Animation task is inconsistent with Quesque and Rossetti who argued the task does not entail representing mental states. They proposed that performance on the task is better explained by low-level processes such as kinematic processing and visual discrimination. Those processes trigger the perception of intention, which is dissociable from the representation of, and reasoning about, covert mental states (Gobbini et al., 2007).

Based upon a hierarchical clustering of meta-analytic results from neuroimaging data in adults, Schurz et al. (2021) proposed that animation tasks represent an intermediate dimension, one that overlaps with affective and cognitive dimensions of social cognition. The association with affective tasks (e.g., measurements of empathy) occurs because the geometric animations depict scenarios that trigger emotional reactions. The overlap with the cognitive dimension occurs because animation tasks also involve inference and reasoning about the mental states of agents. The inclusion of the Animation task in Factor 1 is more closely aligned with the Schurz et al. (2021) analysis of the animation task than with Quesque and Rossetti (2020).

The different loadings of the RMET and Animation tasks are interesting because the tasks appear to share two features in common. One, as just noted, the Animation task presents scenarios that lend themselves to emotionally laden interpretations. In that regard, both tasks are apparently tapping into the affective dimension of ToM. Second, the attribution of mental states and emotions for both tasks have a perceptual basis. In that sense, both tasks appear to tap into social-perceptual processes that infer mental states from nonverbal cues (see Meinhardt-Injac et al., 2020 for a discussion of a social-perceptual ToM component). That is, emotion, thoughts, and intentions are attributed based upon what one observes from geometric movement or the eye regions rather than upon verbal information. Despite their similarities, in the next section we suggest the tasks load onto different factors because only the Animation task has a narrative structure.

Before discussing Factor 1, it is worth highlighting that there is a preliminary, uncertain, quality to it given the small correlations between the instruments that comprise it. In our discussion in the next section, we treat the factor as grounds for future research rather than as conclusive evidence for an underlying structure of ToM.

### 4.1 Narrative comprehension as a cognitive influence on ToM?

Quesque and Rossetti (2020, p. 386) characterized the ability to represent different perspectives as a “core component” of ToM. However, the nature of Factor 1 suggests that their characterization is incomplete in failing to account for the *purpose* for perspective taking. Participants identify and reason about perspectives in order to make sense of what others say and do. Ho et al. (2022, p. 959) state that, “The classic problems (and psychological tasks) used to study ToM require an observer to predict or explain another person’s action.” In all three tasks in Factor 1, participants are interpreting and explaining the actions of agents by inferring mental states within a narrative structure. In that respect, the tasks differ from both the FIMI and RMET. Also in that respect, the three tasks in Factor 1 have in common the requirement that participants reason about mental states within the causal-explanatory framework discussed in the Introduction as a central dimension of ToM.

We propose that the narrative framework of the tasks that comprise Factor 1 helps explain their overlap. The narrative structure of the Strange Stories and Higher-Order False Belief tasks is clear because each task features written text that portrays story-like social scenarios. In contrast, the story structure is inferred by participants from perceptual stimuli in the Animation task. Devine et al. (2016) argued that Strange Stories and Animation tasks both involve inferring mental states to interpret social scenarios. In that characterization (which we share), the Animation task is not simply the low-level recognition of mental states from perceptual stimuli. Rather than passively observing the movement of geometric shapes, participants’ active interpretations appear to invoke a story schema; that is, a memory for how stories are structured into common narrative elements of problem, goal, action, and resolution (e.g., Meadowcroft and Reeves, 1989; see also, Jing and Kirkorian, 2020). Consistent with that interpretation, Nguyen et al. (2019) found that neural activity in regions of the brain associated with narrative and linguistic processing was elicited when adult participants watched nonverbal, story-like animated scenarios of geometric shapes.

Narrative comprehension involves the integration of events within a story while drawing upon one’s own prior experiences and knowledge to interpret them. Participants presumably draw upon their own social knowledge and experiences when explaining what happened in the animated scenarios. For instance, nearly 20% of the participants in this study described a scene in the Animation task (when an agent “surprises” another) by referring to a social ritual—a prank that involves knocking on a door and running away (“ding dong ditch”). Thus, despite the difference in modality—written text or animated motion—all three tasks require integrating a series of events into a coherent structure, while drawing upon one’s own experiences and ToM knowledge to interpret behavior in the scenarios. Only with this prior narrative structure would one extrapolate a social ritual from the movements of two triangles and an empty box.

We refer to Factor 1 as the *protagonist perspective* factor following from Mason and Just (2009). They characterized the overlapping neural regions that support ToM reasoning and narrative comprehension as the *protagonist perspective neural network*. The term refers to the processes used to interpret the action of “any human, animal, or other entity capable of autonomous action that is the focus of a story” (p. 158). The neural network is associated with two neurocognitive processes. One involves monitoring the narrative itself (e.g., monitoring what the protagonist says or does in the context of successive events). The second involves integrating the narrative events in order to make a mentalistic inference to predict and explain action. Both processes are relevant to the tasks in the protagonist perspective factor and neither one is relevant to the two tasks (RMET and FIMI) not included in the factor. In short, the tests in the factor involve reasoning about “perspective” *and* “protagonists” (i.e., the actions of a protagonist in the context of a story-like scenario).

The protagonist perspective factor was positively associated with vocabulary knowledge. The magnitude of the correlation, 0.28, exceeded our threshold of a practically meaningful relationship (below that threshold, there were also very weak correlations with EF measurements too). Vocabulary knowledge is important for accessing background world knowledge that supports narrative inferences and comprehension (e.g., Cromley and Azevedo, 2007). It could be the case that narrative comprehension skills, supported by vocabulary, are a cognitive influence on the tasks in the protagonist perspective factor. Two EF components (working memory and cognitive flexibility) each had a weak but statistically significant relationship with the composite of the z-scores for Animation, Strange Stories, and Higher-Order False Belief. In the Introduction, we discussed that individual differences in general cognitive variables could lead to variability in the expression of ToM knowledge in early adulthood. EF processes such as keeping the details of narrative elements in mind might also exert a weak, but non-negligible, influence on the expression of ToM knowledge on narrative-based tasks in early adulthood (see Cartwright et al., 2020 for a discussion of EF and reading comprehension in early adulthood).

### 4.2 Limitations and future research

There is considerable variation in the response demands of advanced ToM measures, which attenuates correlations and complicates attempts to identify if they conceptually cohere (Devine, 2021). There is a need for research that isolates the influence of task demands, doing so by (a) administering a battery of tasks that share similar conceptual demands, while (b) systematically varying their response demands. Doing so would help disentangle the influence of task-related and conceptual variables on task associations. Limitations in internal consistency among ToM measurements is also a point of caution, and those limitations highlight a need for replication.

Often the internal consistency of ToM measurements is not reported in the literature (Osterhaus and Bosacki, 2022), which complicates basing task selection upon psychometric properties. When possible, we attempted to select improved measures (e.g., the abbreviated RMET, Olderbak et al., 2015). In this study, our goal was to select measures because of their wide usage and their theoretical relevance to the claim that cognitive perspective taking is a core ToM dimension. The instruments are widely used because they are related to conceptually-relevant social and academic variables, an outcome that is evidence of their predictive validity (see Miller, 2022 for a comprehensive review). They are highly representative of the advanced ToM construct.

Researchers routinely use all of the items on a ToM test or use a shortened version based upon a consideration other than reliability (e.g., subtests based upon item content). Practically speaking, researchers usually select a task, and not task items, in ToM research. Following from that practice, our focus was upon taking existing instruments in the field and examining whether they are related, in conceptually meaningful ways, to other ToM instruments and measures of general cognitive variables. Our focus was not on testing the psychometric properties of each one. Nevertheless, we acknowledge that because of relatively weak internal consistency, a ToM instrument could be tapping more than one ToM dimension. A promising alternative approach to the one adopted in this study would involve conducting a factor analysis on individual items (e.g., Osterhaus et al., 2016). In doing so, future research on advanced ToM in adulthood might uncover factors that were not revealed by the approach used in this study.

As we noted in the Introduction, the ToM instruments were chosen for this study because they are widely used, and because they are found in research with both children and adults. While those considerations are consistent with the rationale for the study, the result is that newer, promising measurements were not included. Of particular interest are instruments that have recently refined measurement of the construct in ways that are evident at the neural level. In research with adults, measurements such as the EmpaToM (Kanske et al., 2015) isolate the constructs of ToM and empathy, and find distinct neural profiles associated with each construct (see also, Völlm et al., 2006). ToM instruments associated with specific neural correlates are particularly well-situated to advance a research agenda that aligns the construct, its measurement, and underlying cognitive and neural processes.

Future research is also needed to determine if the protagonist perspective factor represents an underlying structural dimension of ToM. Our sample size was comparable to Warnell and Redcay (2019), but research with a larger sample size would increase confidence in the replicability of the factor. Adding new instruments to the ToM battery – which would add to the need for a larger sample size—is also an area for future research. For instance, the significant relation between the factor and vocabulary clearly signals a role for language skills. Consequently, additional tasks that significantly depart from the verbal demands found in the protagonist perspective tasks (e.g., comprehending verbal narratives, and articulating explanations for characters’ actions) would likely fall outside of the factor. Related, a determinative variable for whether a task “belongs” in a protagonist perspective factor is the extent to which the task requires narrative processing over a series of events. Variability on that dimension should be linked to a task’s inclusion or exclusion in a protagonist perspective factor.

More generally, there is also a need for research on the role that narrative processing plays in ToM task performance. Narrative comprehension involves general inferencing abilities and the integration of information into a coherent, causally-related sequence of events. More research is needed to pinpoint how (or whether) those skills also support ToM reasoning, particularly in light of recent meta-analytic evidence that finds a relation between text comprehension and ToM across the lifespan (Tompkins et al., 2024). Evidence that preschoolers’ ToM is related to their causal sequencing of narratives is a promising step in that regard (Tompkins et al., 2020).

We only included one measurement in this study, the RMET, identified by researchers as representative of an affective dimension of ToM (e.g., Schurz et al., 2021). Therefore, our findings do not speak to that hypothesized feature of the ToM construct. It is worth asking in future research if narrative-like scenarios designed to elicit perspective taking about affective states might align more closely with the RMET or with tasks associated with the protagonist perspective factor. In other words, is the mental state content that participants attribute (affective or cognitive) more determinative than the purpose of those attributions (to predict and explain action in a narrative structure) when assessing task coherence?

Our study was also limited to a restricted age range. In doing so, we avoided conflating individual and developmental differences. But the results do not speak to whether there is continuity or discontinuity in ToM development. Recall from the Introduction that we intentionally chose instruments in this study that are also used in child development studies. In doing so, our findings pinpoint a question for future research. Osterhaus et al. (2016) reported a “social reasoning” factor in children ages 8- to 10-years that was comprised of Higher-Order False Belief measurements, a subset of items from the Strange Stories task, and a child version of the RMET. Whether the different findings with respect to the RMET reflect developmental discontinuity or methodological differences between studies requires further research. As we discussed in the Introduction, it could be the case that ToM in childhood is less differentiated than it is in adulthood; consequently, distinctions between measurements such as Higher-Order False Belief and RMET may be found in adults but not in childhood. In sum, this study provides evidence for how the instruments align in adulthood. Evidence for continuity and discontinuity in the structure of ToM awaits research with different age groups.

Alternatively, an expanded focus that includes older participants would also inform how (or whether) response demands on cognitive abilities influence task coherence. For instance, there is evidence that age-related declines in ToM are tied to declines in EF rather than ToM reasoning (Cho and Cohen, 2019). When task-related EF demands are reduced, age-related deficits in ToM performance are also reduced. The point, more generally, is that the likelihood of coherence could be tied to moderators such as age that influence whether demands mask conceptual knowledge. Similar concerns extend to neurodivergent individuals for whom performance factors may substantially obscure underlying conceptual competencies (see Montgomery et al., 2023 for a discussion). The extent to which the findings reported in this study are generalizable to other populations awaits further research.

## 5 Conclusion

In sum, this research investigated the nature of the ToM construct by asking if commonly used advanced ToM measures cohere in early adulthood. We also addressed a need in the literature for a comprehensive analysis of the influence of vocabulary, EF, and SES on adult ToM. We administered a battery of tasks to over 200 participants who, collectively, mirrored race and ethnicity proportions in the U.S. The Strange Stories and Higher-Order False Belief task exhibited a meaningful association. We also found evidence for what we termed a protagonist perspective factor. The factor is related to general vocabulary skills, indicating that the influence of language on ToM extends beyond childhood. The factor also implicates narrative comprehension skills as an additional cognitive influence on ToM, and that suggestion warrants future research.

## Data Availability

The raw data supporting the conclusions of this article will be made available upon reasonable request.
